# The Rise of Adaptive Platform Trials in Critical Care

**DOI:** 10.1164/rccm.202401-0101CP

**Published:** 2024-01-25

**Authors:** The PRACTICAL, PANTHER, TRAITS, INCEPT, and REMAP-CAP investigators

**Keywords:** platform trials, adaptive design, critical care

## Abstract

As durable learning research systems, adaptive platform trials represent a transformative new approach to accelerating clinical evaluation and discovery in critical care. This Perspective provides a brief introduction to the concept of adaptive platform trials, describes several established and emerging platforms in critical care, and surveys some opportunities and challenges for their implementation and impact.

## Adaptive Platform Trials as Learning Research Systems

During the past half century, the randomized clinical trial has assumed a fundamental role in advancing practice and improving outcomes in medicine and in critical care (1). Trials provide the most rigorous approach for causal inference about treatment effects and thus offer the best means to test the clinical effectiveness of new (and old) treatments, strategies, and practices in the ICU.

Yet, for all their impact, clinical trials are difficult and arduous to perform. They are expensive, time-consuming, complex, and subject to governance and regulatory barriers. Massive amounts of time and effort are required to launch and conduct trials. Multicenter trials require large networks of sites governed by legal contracts, data-sharing agreements, and funding conditions. Investigators may find it easier to set aside long-term investments required for fundamental improvements in trial conduct (i.e., automated data collection from electronic medical records) to “get the trial done.” Given issues with time and capacity, collection of mechanistic data and biological samples during trials, which could help in understanding positive and negative trial results, is less common and represents a missed opportunity. And, after all the time and effort invested in running trials, too often trial results produce inconclusive results (2), neither definitely ruling out benefit nor definitively ruling it in, partly because traditional fixed sample size designs require an “educated guess” of treatment effect and partly because it is challenging to build a network of sufficient size to generate sample sizes that are adequate for the detection of small treatment effects in a timely manner.

To overcome these barriers, we need a new approach to conducting clinical research and clinical trials, one that adopts a long-term vision for a durable integrated collaborative research system. Such a system would build on the initial work required to set up a single trial—investigator network, governance and regulatory structures, data management systems—to create a long-term foundation for collaboration, discovery, and improvements in care. Methodological expertise and innovation can be layered on top of this foundation and applied across multiple trials to fundamentally enhance operations and insights. Operating a research system over the long term affords the opportunity to learn what does and does not work and to progressively improve efficiency and effectiveness. Future trials within the system could leverage these methods and learning for greater success, efficiency, and insights. Indeed, information about patient characteristics and outcomes can be literally “borrowed” to enhance the precision of treatment effect estimates in later trials. This research system and culture of learning through experience manifests in adaptive platform trial designs that use information accumulating in the trial to ensure that the trial operating characteristics (e.g., sample size) afford clear conclusions to inform clinical decisions. Adaptive platform trials aim to be durable learning research systems (3–5). Larger upfront investment in terms of time and money may be required for an adaptive platform trial, but, over the long term, provide a foundation for more efficient collaboration, discovery, and improvements in care (6).

Adaptive platform trials have been around for well over a decade (7), but gained wide attention during the coronavirus disease 2019 (COVID-19) pandemic, with demonstrated success in the rapid evaluation of multiple therapies (*see* REMAP-CAP below). The pandemic also provided demonstrative potential for multiplatform trials, whereby the same intervention protocol was applied across multiple platforms and analyzed as a single trial to maximize global coverage and accelerate trial conclusions (8). Building on this experience, several adaptive platform trials are now being initiated to investigate new treatments and strategies for critical illness. Here we briefly summarize them, highlighting key areas of focus and unique methodological features (Table 1).

**Table 1.** Definitions of Terms Employed in Different Adaptive Platform Trials

Term	Definition
Adaptive trial	A trial design that includes the possibility of prespecified conduct of the trial based on information accumulating in the trial. Design features that can be adapted include sample size, adaptive stopping and arm dropping, randomization ratio, available interventions or arms, eligibility criteria, and outcomes.
Adaptive platform trial	An adaptive platform trial studies “multiple interventions in a single disease or condition in a perpetual manner, with interventions allowed to enter or leave the platform on the basis of a decision algorithm” (4, 19).
Domain	A “therapeutic area” or set of interventions under study that target the same disease mechanism or pathway using a similar overarching approach (e.g., different lung-protective ventilation strategies) with a common control group. Domains function as adaptive platform trials in their own right; patients can be enrolled and randomized in multiple domains simultaneously to facilitate investigation of different therapeutic regimens.
State and stratum	State and stratum are variables used to categorize patients for the purpose of specifying eligibility for a domain or as a grouping for subgroup-specific treatment effects (i.e., treatment effect estimated within a specific state or stratum). The two terms differ with respect to the effect of time. State can vary over time; a patient can be randomized in one state in one domain and then subsequently randomized in a different state in a different domain. By contrast, stratum is assigned based on patient characteristics ascertained at baseline; the stratum to which a patient belongs is a fixed characteristic that does not change over time.
Treatable trait	Treatable traits can be defined as measurable patient characteristics that are proximate determinants of clinical outcomes (e.g., organ dysfunction or death) and are plausibly modifiable with interventions. Such treatable traits in critically ill adults are conceptualized as agnostic of critical illness syndromes. Stated differently, the same treatable trait can be found in different critical illness syndromes such as sepsis, pancreatitis, trauma, and acute respiratory distress syndrome. In the TRAITS trial, critical illnesses are grouped into treatable traits identified by combining a biomarker and a bedside clinical variable of organ dysfunction. Thus, trait identification is unaffected by issues associated with diagnostic test performance and probabilistic allocations. Critically ill patients can have more than one trait concurrently.

*Definition of abbreviation*: TRAITS = Evaluation of interventions linked to treatable traits in acute critical illness in adults to enable precision medicine: Data enabled Bayesian adaptive platform randomized clinical trial with embedded biological characterization.

## Established and Emerging Adaptive Platform Trials

### REMAP-CAP

The REMAP-CAP (Randomized Embedded Multifactorial Adaptive Platform trial for Community Acquired Pneumonia) is an established pioneer in adaptive platform trials (www.remapcap.org). Originally conceived after the influenza H1N1 pandemic in 2009, REMAP-CAP has recruited more than 13,000 patients with severe community-acquired pneumonia since 2016 from more than 300 hospitals worldwide, on every continent. It is an ongoing, investigator-initiated platform trial that continues to evaluate a variety of interventions for hospitalized patients with severe respiratory infections, including COVID-19. Since inception, it has reported results from nine domains, including IL-6 receptor antagonists, vitamin C, angiotensin-converting enzyme inhibitors, and simvastatin for COVID-19 (9–11), and has evaluated many additional interventions or combinations of interventions, structured within multiple treatment domains. Nearly 50% of patients randomized in REMAP-CAP have been randomized to more than one domain, highlighting the potential information gain facilitated by a platform in which a single participant contributes information about treatment effect across multiple trials.

REMAP-CAP uses a Bayesian hierarchical analytical model that includes patient-specific strata such as patient age (pediatric and adult) and infecting pathogen (severe acute respiratory syndrome coronavirus 2 [SARS-CoV-2], influenza, and bacteria) and patient-specific factors to inform states, such as severity of illness at the time of randomization, forming units of analysis that reflect these strata and state combinations. Adaptations within the design include, among others, response adaptive randomization to weight randomization ratios according to emerging data about treatment effects being generated in the trial, predefined stopping rules that are informed by extensive simulations without a prespecified sample size, updated strata and state combinations, and, for the COVID-19 pandemic, an updated primary outcome (organ support–free days at Day 21). It continues to evaluate a variety of interventions for children and adults with severe community-acquired pneumonia.

### PRACTICAL

PRACTICAL (Platform of Randomized Adaptive Clinical Trials in Critical Illness; www.practicalplatform.org) is a new platform trial studying interventions for acute hypoxemic respiratory failure across multiple therapeutic domains, with an early focus on respiratory support strategies and corticosteroids. PRACTICAL aims to create a pipeline of trials in acute hypoxemic respiratory failure across multiple phases of investigation (pilot and feasibility trials and phase II, III, and II/III trials). The statistical model employs a physiology-based precision medicine design, permitting different adaptations among four severity states (nonintubated, low respiratory system elastance, high respiratory system elastance, extracorporeal life support required) to prospectively account for heterogeneity of treatment effect according to the severity of hypoxemic respiratory failure (12). Domains function relatively independently with separate primary endpoints. Some domains in PRACTICAL are observational studies, enabling specialized data collection (e.g., respiratory waveforms and patient–ventilator interaction) to be linked to treatment effects observed in other domains. PRACTICAL is focused on developing methods for integrating prospective discovery of heterogeneity of treatment effect into trial design adaptations. PRACTICAL is already actively recruiting in the United States and Canada, and additional sites in Argentina, Europe, Saudi Arabia, Australia, and New Zealand are joining in the very near future.

### PANTHER

The overall aim of PANTHER (Precision Medicine Adaptive Platform Network Trial in Hypoxemic Acute Respiratory Failure) is to accelerate the development of pharmacological therapies for acute hypoxemic respiratory failure and acute respiratory distress syndrome (ARDS) by establishing an international phase II precision medicine adaptive platform trial (https://panthertrial.org). PANTHER will examine patients with hyper- and hypoinflammatory phenotypes of ARDS (13) to answer the question whether, compared with usual care alone, simvastatin or an additional biologically targeted intervention (to be determined) increases organ failure–free days at Day 28 in patients with ARDS as defined by the newly published global definition (14). PANTHER will use a Bayesian adaptive multiarm trial design with predefined triggers for efficacy and futility. Regular adaptive analyses will enable investigators to identify differential treatment responses across phenotypes by examining treatment effect in phenotype strata and stopping arms in the case of evidence of futility or efficacy. Inflammatory phenotype will be prospectively determined in real time before randomization using a validated algorithm based on plasma IL-6, soluble tumor necrosis factor receptor-1, and bicarbonate (15). PANTHER will also collect extensive biological samples, allowing further mechanistic studies to be conducted in parallel with the conduct of the platform trial, potentially allowing further phenotypes and interventions to be added to the platform over time.

### INCEPT

INCEPT (Intensive Care Platform Trial) is a randomized, multifactorial, adaptive platform trial planned to enroll acutely admitted adult ICU patients within the CRIC (Collaboration for Research in Intensive Care) network. Interventions will be assessed in multiple domains, including dosing strategies for prophylactic low molecular weight heparin, use of albumin in shock, and continuous glucose monitoring. INCEPT uses adaptive stopping, arm dropping, and allocation ratios based on predefined criteria, simulations, and sequential Bayesian analyses (16). The primary and guiding outcomes will be based on a core outcome set for general ICU patients developed in the INCEPT group together with patients, family members, clinicians, and researchers (17). These stakeholders will also be involved in planning consent processes and prioritizing interventions. A data model containing domain structure and electronic case report forms will automatically capture data from electronic patient records. A funding model was developed with hospital managers to supplement external philanthropic funding. INCEPT is preceded by the EMPRESS (Empirical Meropenem versus Piperacillin/Tazobactam for Adult Patients with Sepsis) adaptive trial (https://www.cric.nu/empress/) using parts of INCEPT infrastructure and methodology. INCEPT will commence in 2024 and is expected to run in many ICUs, initially mainly in Northern Europe.

### TRAITS

TRAITS (Evaluation of interventions linked to treatable traits in acute critical illness in adults to enable precision medicine: Data enabled Bayesian adaptive platform randomized clinical trial with embedded biological characterization) is a research program aimed at enabling precision medicine to improve outcomes for critically ill adult patients (https://traits-trial.ed.ac.uk). The key design features include a data-enabled, embedded, multiarm, umbrella, adaptive platform trial within a Bayesian analytic framework (ISRCTN Registry no. 82395639). The primary objective is to evaluate the efficacy of experimental treatments on prespecified outcomes in critically ill adult patients who meet the eligibility criteria for a specific syndrome-agnostic treatable trait (defined in Table 1) compared with usual care. All analyses will be performed using Bayesian ordinal regression models to calculate treatment effects of active interventions (vs. control), and these analyses will adjust for prespecified prognostic variables, with priors informed by accruing trial data as well as external information. For sequential learning with interim adaptive analyses, the trial has prespecified intermediate outcomes for each trait (stage 1 analyses). When interventions reach the prespecified maximum sample size or meet the criteria for efficacy or futility, based on the posterior probability of clinically important efficacy, in stage 1 analyses, the treatment effect will be reported for the trial primary outcome of organ support–free days to Day 21 (stage 2 analyses). The data-enabled design uses existing data-capture frameworks within the National Health Service in Scotland to streamline the data collection and create models for efficient data flow in clinical trials. The embedded biological sampling uses systems immunology approaches (18) to study the mechanisms of intervention effect and enable discoveries such as new treatable traits, biological heterogeneity of treatment effect, and biomarkers.

## Challenges for Adaptive Platform Trials in Critical Care

Each of these research systems will grow and develop over time to execute clinical trials more cost-effectively and efficiently (6), ultimately achieving higher-quality, more informative trial results to guide clinical decisions for patients and families. Adaptive platform trials should not, however, be viewed as a panacea. Despite the strengths of platforms and the opportunities they present, they have relevant weaknesses and face important threats (Table 2). Some of the methods and analytical strategies remain unfamiliar to the broad clinical community, potentially hampering knowledge translation. The regulatory and funding mechanisms created for traditional clinical trials must evolve to ensure that adaptive platform trials can maximize the potential benefits they offer to investigators, the clinical community, and patients.

**Table 2.** Considerations for Adaptive Platform Trials in Critical Care

Category	Consideration
Strengths	• Capacity to leverage shared, standardized governance, methods, and analytical infrastructure across multiple trials for long-term operational efficiency (easier to conduct additional new trials) and quality (infrastructure will be rigorously evaluated and improved over time) • Use of a common or shared control group can significantly reduce net sample size requirement across multiple interventional trials • When patients are randomized in multiple domains, can evaluate the effects of combinations of interventions (including interactions between interventions) • Response-adaptive randomization can help ensure the probability of receiving treatment aligns with the most current degree of equipoise as to benefit or harm • Potentially fewer legal and contractual interinstitutional agreements (though the individual agreements are more complex) • Ability to quickly incorporate new available treatments and/or new forms of the disease under investigation (in the case of a pandemic) • Incentivizes long-term collaboration with trial methodologists, which provides opportunity for innovations in trial conduct
Weaknesses	• Adaptive designs are complex, require extensive simulation and preplanning, and are still unfamiliar to many stakeholders • Infrastructure and capacity require years to build; number of research questions requiring urgent attention may exceed capacity of research system and network (e.g., pandemic context) • Less flexibility in trial design for individual investigators • Response-adaptive randomization (a type of adaptation whereby randomization ratio is modified according to probability of benefit) may increase sample size requirements under certain conditions • Regulatory complexity for participating sites with master protocols, ethics applications, amendments
Opportunities	• Ability to learn and improve trial conduct over time • Long-term commitment to platform function motivates strategic investments in operational improvements and efficiencies (i.e., automated data capture) • Apply methodological innovations (i.e., detection of heterogeneous treatment effects) across multiple trials • Embedding adaptive platform trials within healthcare systems can enable learning healthcare systems as a continuous quality-improvement mechanism • Patient and public involvement and engagement could promote social understanding of long-term public health benefit of durable research systems
Threats	• Platform operation creates some operational costs not associated with individual clinical trials • Differences in patients over time create challenges for comparisons (e.g., nonconcurrent controls or population time drift) • Difficult to obtain platform-level funding; to date, funding agencies prefer traditional model of investing in one trial at a time • Difficult to organize multinational funding • Sample size adaptation may be (incorrectly) perceived as biased through comparison versus traditional concerns about “early stopping for benefit” • Transparent governance is critical to ensure fairness, equity, accessibility, rigor, and openness • Critical to communicate open posture to enable new investigators to join and make use of the platform • Ensuring timely and genuinely informed consent for patients or substitute decision-makers presented with the opportunity to be randomized to multiple interventions across multiple domains • Challenging to ensure appropriate academic recognition for all participating investigators given the substantial team effort involved in running these trials; academic institutions may need to evolve to place higher value on collaborative efforts

A commitment to collaboration is perhaps the crucial ingredient in the continued progress and long-term success of these research systems. These collaborations must develop on several fronts. First, “within-platform” collaboration is essential. Clinical research and clinical trials in general are team sports, but platform trials in particular require investigators to surrender some degree of autonomy to ensure that different trials and domains within the platform fit together seamlessly. Second, “between-platform” collaboration is advantageous. When appropriate opportunities arise, it may be more efficient for platforms to leverage the networks and expertise represented by other platforms to expand the reach of their trials, as exemplified by the concept of the multiplatform trial. Platforms should also maintain strong lines of communication to minimize competition or conflict between their operations. Working together to align data collection and outcomes will maximize the long-term value of information in platforms and facilitate multiplatform trial collaborations. To this end, REMAP-CAP, PANTHER, and PRACTICAL have worked closely to align and standardize their biological sampling procedures. Third, “beyond-platform” collaboration is crucial. These tight-knit research communities must proactively work to be outward-looking and welcoming to those interested in joining the platforms. Investigators early in their careers should find platform trials an especially beneficial development; by working within platforms, they are able to leverage the trial infrastructure already created to undertake their studies and trials and answer their research questions within a supportive and well-mentored research environment. Platforms will need to consider strategies to communicate the invitation to join their research system to the broader clinical and scientific community in critical care.

In sum, adaptive platform trials hold a great deal of potential for the field of critical care. To make the most of that potential, a great deal of time, effort, and funding must be invested, and a deep commitment to collaboration must be sustained.
